# Excessive hospitalization of patients with seizures in the Germany prehospital emergency system: a retrospective cohort study

**DOI:** 10.1038/s41598-022-15115-8

**Published:** 2022-06-27

**Authors:** Kristina E. Fuest, Claudia Hofberger, Marco Lorenz, Bernhard Ulm, Karl-Georg Kanz, Manfred Blobner, Stefan J. Schaller

**Affiliations:** 1 Technical University of Munich, School of Medicine, Klinikum rechts der Isar, Department of Anesthesiology and Intensive Care, Munich, Germany; 2 Charité – Universitätsmedizin Berlin, corporate member of Freie Universität Berlin and Humboldt-Universität zu Berlin, Department of Anesthesiology and Operative Intensive Care Medicine (CVK, CCM), Berlin, Germany; 3 Technical University of Munich, School of Medicine, Klinikum rechts der Isar, Department of Trauma Surgery, Munich, Germany

**Keywords:** Outcomes research, Neurological manifestations, Neurological disorders

## Abstract

Seizures are a common reason for calling emergency medical services. A lack of guidelines on prehospital treatment in Germany leads to high transportation rates and reduced confidence in decision making by professionals. Our aim was to investigate the reasons for hospitalization and evaluate their necessity. A retrospective analysis of all emergency medical services records in Munich, Germany was performed in order to examine the reasons for hospitalization of patients with seizures and to evaluate their trajectory following admission to a university hospital. 8882 records were analyzed with 415 records reporting seizures (4.9%). Primary endpoint was transportation to hospital. In 380 cases (92%) patients were transported, of which 177 patients (47%) had known epilepsy; 35 patients (8%) were left at scene. Older patients and patients with higher amounts of administered medication at the scene were hospitalized significantly more often (p = 0.032 and p = 0.004, respectively). Median hospital length of stay was 1 night [IQR 1–2]. In patients with out-of-hospital seizures, high hospital transportation rates were evident, most of which could be considered as not indicated. One possible reason is the lack of guidelines in Germany, which leads to uncertainty among medical staff. This results in distress for the patients, their caregivers and consequently high costs.

## Introduction

Seizures rank among the top 10 reasons for calling the emergency medical services^1–3^. Status epilepticus is a life-threatening condition with a mortality rate of about 20%^4^, but it only occurs in approximately 2–7% of patients who present prehospitally with seizures^1,2,5,6^. In most cases the seizure has already ceased by the time the emergency medical service arrives. Nevertheless, 75–88% of these patients are transported to hospital^1,2^. Guidelines for management of patients with a prehospital seizure are rare and many aspects of management are based on expert opinion alone^7^. In Germany, there are no official guidelines for treating seizures in prehospital emergency medicine. Regional, non-official guidelines focus on the treatment of prehospital status epilepticus and do not cover self-resolving seizures^8,9^. In the United Kingdom (UK) “Ambulance Services’ Clinical Practice Guidelines” form the basis for UK paramedic training^7^. These guidelines advise transport to a hospital for all patients with major ABCD-problems (Airway, Breathing, Circulation, Disability), serious head injury, status epilepticus or a suspected underlying infection. Furthermore, patients with either their first seizure or multiple seizures and those, who are difficult to monitor, should be taken to an emergency department. For patients with known epilepsy the guideline recommends managing these patients at home, if they make a full recovery of consciousness, are not at risk and can be adequately supervised^7,10^. Even with these recommendations available, a lot of patients still get transported to hospital^1^. This is most likely caused by a lack of confidence of the EMS (Emergency Medical Service) staff due to either insufficient training, guidelines^1,11,12^ or missing information from the patient’s history or previous treatment. Although data shows that patients with a known seizure disorder who are not transferred to hospital have a minimal risk of recurrence or adverse outcome^13^, no outcome data is available on patients, who were hospitalized. In this study we analyzed EMS records of patients with prehospital seizures, supplemented with in-hospital data. We evaluated the patient’s condition, the reason for transportation and whether there is an excessive hospitalization of patients that formally meet the criteria to be left at the scene^7,14^.

## Results

**Figure 1:**
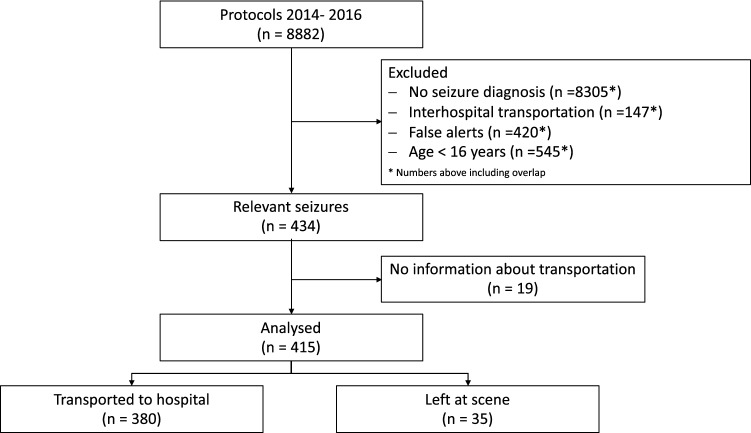
STROBE diagram.

A total of 8882 emergency physician records were available for the years 2014–2016 and were included in the analysis. Seizures represented 4.9% of all emergency deployments. We excluded 8467 patients. A total of 415 records were analyzed for this study (for further details see Fig. 1). Patient characteristics are presented in Tables 1 and 3. 15% of the included patients had a documented pre-existing neurological condition. Patients with a history of epilepsy were significantly younger (median 42 [IQR 29–61], p = 0.002). No other significant difference in patient characteristics could be found between patients with or without a diagnosis of epilepsy.

**Table 1:** Characteristics of all patients stratified by known and unknown diagnosis of epilepsy.

	All patients, n = 415	Patients without history of seizure/epilepsy, n = 209	Patients with history of seizure/epilepsy, n = 206	p-Value
Sex (female)	181 (44%)	88 (42%)	93 (45%)	0.599
Age (median (IQR))	47 (31–65)	51 (34–69)	42 (29–61)	0.002
Pre-existing neurological condition	63 (15%)	25 (12%)	38 (18%)	0.088
Seizure relating alcohol- or substance abuse	51 (12%)	28 (13%)	23 (11%)	0.587
GCS at EMS arrival (median (IQR))	14 (9–15)	14 (8–15)	14 (9–15)	0.095
GCS at handover (median (IQR))	14 (12.5–15)	14 (12–15)	15 (13–15)	0.100

“Pre-existing neurological condition” includes brain tumor and metastases, intracranial hemorrhage and malformations as well as infantile brain damage and stroke.

*GCS* Glasgow-Coma-Scale, *EMS* Emergency Medical Service, *n/a* not available.

### Hospital transport

Table 2 lists the reasons for transport to the hospital. In 155 cases, no indication was documented (39.5%). In total 380 patients (92%) were transported to a hospital and 35 were left at the scene (8%). In 16 of these cases the patients refused transportation, in 17 cases the emergency physician decided to leave the patient at the scene and in two cases the patients were left due to predefined therapy limitation. Of the patients left at the scene 29 had a known history of seizures (83%). The median GCS at EMS arrival was 14 [IQR 9–15] in all patients. In patients with a known history of seizures or epilepsy GCS had increased by the time of handover in the hospital (15[IQR 13–15] (Table 1).

**Table 2:** UK Criteria for home management and corresponding reasons for hospitalization given by the emergency physicians in Munich.

UK guidelines for home-management	Reason for transportation given by Munich emergency physicians
Full recovery	Status epilepticus (n = 52)
	Subsequent seizure (n = 35)
Known epilepsy	GCS ≤ 14 at handover (n = 114)
	Still seizing at EMS arrival (n = 77)
Not at risk	Possible TBI (n = 17)
	Pre-existing neurological condition (n = 63)
Adequately supervised	Seizure relating to alcohol or substance abuse (n = 51)
	Possible infection/fever (n = 12)
	Benzodiazepine administered by EMS (n = 122)
	No reason documented (n = 155)

“Pre-existing neurological condition” includes brain tumor and metastases, intracranial hemorrhage and malformations as well as infantile brain damage and stroke.

*TBI* Traumatic Brain Injury, *GCS* Glasgow-Coma-Scale, *EMS* Emergency Medical Service, *n/a* not available.

In the univariate analysis gender, GCS at EMS arrival, first occurrence of a seizure, abuse of alcohol, the number of medications administered by the prehospital emergency team and, if the patient was still convulsing or had a status epilepticus showed significant influence on the hospital transportation rates (Table 3).

**Table 3:** Hospital transportation rates and potential influencing factors for all patients and stratified by hospital.

Hospital transportation				Destination of transport	
	Left at scene, n = 35	Hospital transportation, n = 380	p-value	To our specific university hospital, n = 92	To other hospital, n = 288
Sex (female, n (%))	22 (63)	159 (42)	0.026	38 (41.3)	121 (42.0)
Age (median (IQR))	42 (30–61)	47.27 (31.1–65.3)	0.501	47 [30, 63]	47 [31, 66]
**Initial survey and medical history**					
Body temperature (mean (SD))	36.4 (0.6)	37.2 (1.0)	0.084	37.2 (1.1)	37.2 (1.0)
Missing	31 (89)	312 (82)	0.463	79 (85.9)	233 (80.9)
GCS at EMS arrival (median (IQR))	15 (14–15)	14.00 (9.0–15.0)	< 0.001	14 [9, 15]	14 [8, 15]
First occurrence of a seizure, n (%)	6 (17)	203 (53)	< 0.001	40 (43.5)	163 (56.6)
Pre-existing cerebral damage, n (%)	6 (17)	57 (15)	0.927	17 (18.5)	40 (13.9)
Alcohol abuse, n (%)	0 (0)	51 (13)	0.041	15 (16.3)	36 (12.5)
Existing injuries, n (%)	2 (6)	49 (13)	0.332	15 (16.3)	34 (11.8)
Still convulsing, n (%)	1 (3)	76 (20)	0.023	22 (23.9)	54 (18.8)
Relevant comorbidities, n (%)	4 (11)	116 (31)	0.029	48 (52.2)	158 (54.9)
Uncontrolled urine leakage, n (%)	3 (9)	44 (12)	0.796	11 (12.0)	33 (11.5)
Signs of infection, n (%)	0 (0)	9 (2)	0.753	1 ( 1.1)	8 ( 2.8)
Follow-up seizure, n (%)	1 (3)	34 (9)	0.356	7 ( 7.6)	27 ( 9.4)
**Type of seizure, n (%)**			0.063		
Generalized	25 (76)	325 (89)		80 (89.9)	245 (88.8)
Simple focal	3 (9)	19 (5)		4 (4.5)	15 (5.4)
Focal with altered consciousness	5 (15)	21 (6)		5 (5.6)	16 (5.8)
Traumatic brain injury, n (%)	3 (9)	14 (4)	0.342	5 (5.4)	9 (3.1)
Postictal state, n (%)	11 (31)	243 (64)	< 0.001	55 (59.8)	188 (65.3)
Status epilepticus, n (%)	0 (0)	52 (14)	0.038	12 (13.0)	40 (13.9)
Psychogenic, n (%)	3 (9)	13 (3)	0.291	4 ( 4.3)	9 ( 3.1)
Pre-existing dementia	0 (0)	10 (2.6)	0.692	0 (0)	10 (3.5)
**Initiated therapy**					
Glucose, n (%)	0 (0)	12 (3)	0.589	2 ( 2.2)	10 ( 3.5)
Induction of anesthesia & intubation, n (%)	0 (0)	10 (3)	0.692	4 (4.4)	6 (2.1)
Patients with administered medication (without liquids), n (%)	7 (20)	166 (44)	0.011	34 (37.0)	132 (45.8)
if administered (median (IQR))	1.00 (1.0–1.0)	1.00 (1.0–2.0)	0.085	1 [1, 2]	1 [1, 2]
Documented reason for transportation, n (%)	–	238 (62.6)	–	64 (69.6)	174 (60.4)

Data are median and interquartile range or n. Reference for sex is female.

“Pre-existing cerebral damage” includes brain tumor and metastases, intracranial hemorrhage and malformations as well as infantile brain damage and stroke; CI, Confidence Interval; Logistic regression was calculated for univariate significant variables for patients without substance abuse or without status epilepticus, odds-ratios and p values are presented.

*IQR* Interquartile Range, *GCS* Glasgow-Coma-Scale, *EMS* Emergency Medical Service.

### Patients with history of seizure

A history of seizures/epilepsy was present in 206 patients and 177 (86%) of those were transported to a hospital. A postictal state existed in 61% which is on its own not considered a sufficient indication for transportation according to the UK guideline.

### Patient outcomes

**Figure 2:**
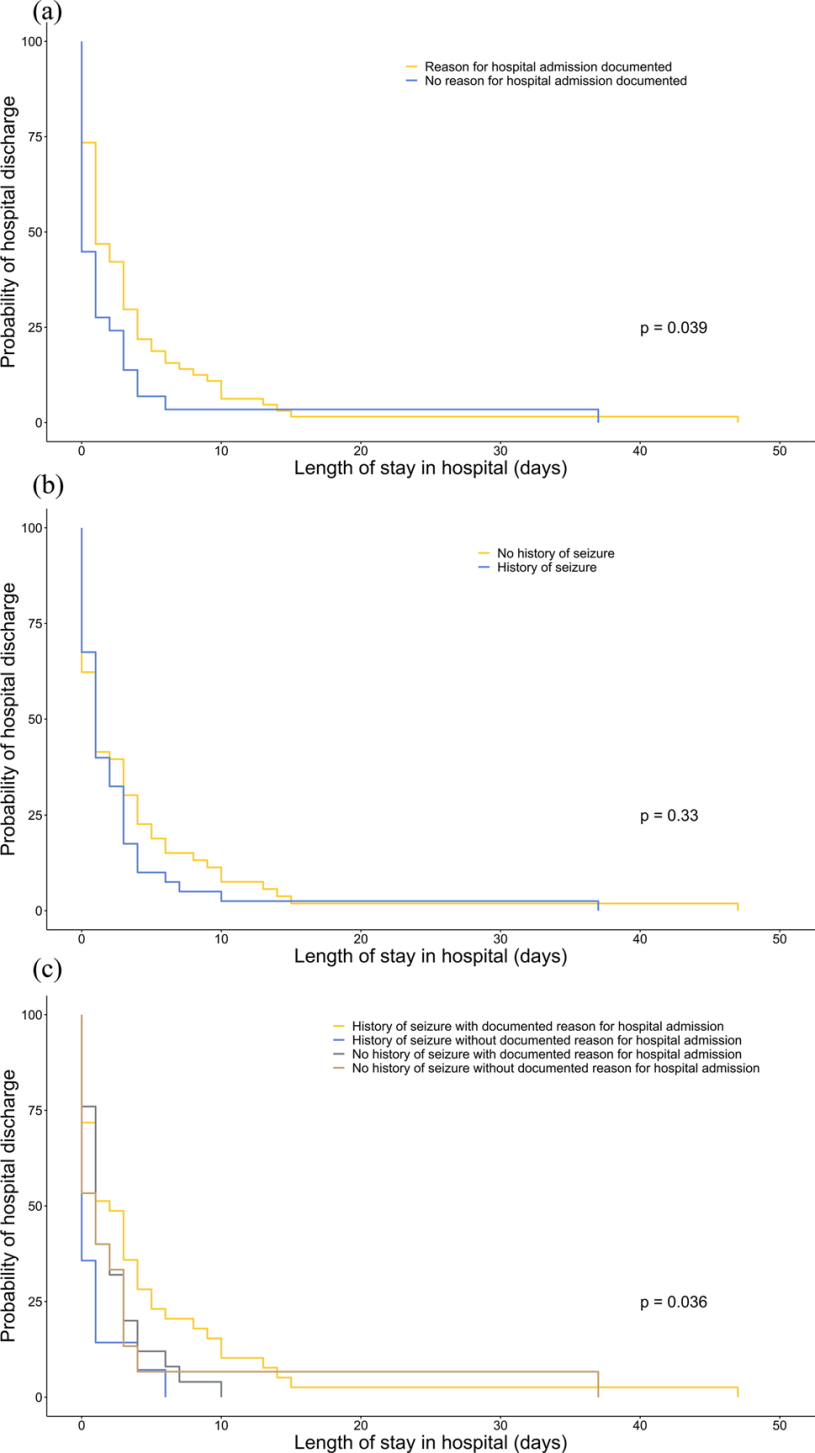
Probability of hospital discharge. Probability of hospital discharge stratified by a documented transportation reason (**a**) and history of seizure/epilepsy (**b**) presented as Kaplan–Meier-Curves. (**c**) Shows the combination of documented reason for admission and history of seizures. Yellow line indicates patients with a documented reason for hospital transportation (**a**) or no history of seizure/epilepsy (**b**); blue line indicates patients without documented reason (**a**) or history of seizure/epilepsy (**b**). Please see Supplementary Table 1 for possible transportation reasons as documented by the attending emergency physician.

104 patients were transported to the university hospital “Klinikum Rechts der Isar”. For 92 patients, data was available via the hospital patient management system. Patients brought to hospital did not differ in their characteristics from those transported to other hospitals in the region (Table 3). Patients remained at the hospital for a median of 1 night [IQR 1–2]. The group of patients with no documented reason for transportation stayed a significantly shorter time (p = 0.039) (Fig. 2a). There was no significant difference in the hospital length of the stay between patients with or without a history of seizures/epilepsy (p = 0.33) (Fig. 2b). Kaplan–Meier-curves for the combination of both factors showed a significantly higher probability of hospital discharge for patients with a history of seizures and no documented reason for transportation by the emergency physician (p = 0.036, Fig. 2c). These results were confirmed in a post-hoc-analysis between the groups admitted to the hospital: with and without a reason for transportation with a history of seizures/epilepsy (p = 0.049).

None of the patients being transported to our emergency department died in the hospital. Of all transported patients 33 (35.5%) were only seen in the outpatient centre and not hospitalized (Table 4). Older patients and patients with higher amounts of administered medication at the scene were hospitalized significantly more often (p = 0.032 and p = 0.004, respectively). First occurrence of a seizure had no significant influence on hospitalization (p = 0.628). If there was no documented reason for transport, patients were significantly more likely to only require outpatient care (p = 0.019).

**Table 4:** Comparison of patient characteristics in correlation with hospital length of stay.

	Outpatient clinic, n = 33	Hospitalisation for ≤ 24 h, n = 21	Hospitalisation for ≥ 24 h, n = 38	p-value
Sex (female, n(%))	12 (36.4)	6 (28.6)	20 (52.6)	0.154
Age (median (IQR))	40 [29, 50]	46 [29, 59]	57 [35, 76]	0.026
**Age categories**				0.032
≤ 50	24 (72.7)	11 (52.4)	13 (34.2)	
51–65	5 (15.2)	6 (28.6)	14 (36.8)	
> 65	4 (12.1)	4 (19.0)	11 (28.9)	
**Initial survey & medical history**				
Body temperature (mean (SD))	38 (1)	36 (1)	37 (1)	0.358
GCS at EMS arrival (median (IQR))	14 [13, 15]	9 [7, 15]	14 [10, 15]	0.113
GCS at handover (median (IQR))	15 [14, 15]	12 [8, 14]	14 [14, 15]	0.025
First occurrence of a seizure, n(%)	20 (60.6)	10 (47.6)	22 (57.9)	0.628
Pre-existing cerebral damage, n(%)	4 (12.1)	2 (9.5)	11 (28.9)	0.092
Alcohol abuse, n(%)	4 (12.1)	6 (28.6)	5 (13.2)	0.222
Existing injuries, n(%)	6 (18.2)	4 (19.0)	5 (13.2)	0.788
Psychogenic, n(%)	4 (12.1)	0 (0.0)	0 (0.0)	0.024
Still convulsing, n(%)	5 (15.2)	5 (23.8)	12 (31.6)	0.270
Uncontrolled urine leakage, n(%)	3 (9.1)	2 (9.5)	6 (15.8)	0.636
Follow-up seizure, n(%)	1 (3.0)	2 (9.5)	4 (10.5)	0.460
**Type of seizure, n(%)**				0.338
*generalized*	26 (86.7)	21 (100.0)	33 (86.8)	
focal	1 (3.3)	0 (0.0)	3 (7.9)	
focal with altered consciousness	3 (10.0)	0 (0.0)	2 (5.3)	
Relevant comorbidities, n(%)	15 (45.5)	10 (47.6)	23 (60.5)	0.400
Traumatic brain injury, n(%)	2 (6.1)	1 (4.8)	2 (5.3)	0.977
Postictal state, n(%)	18 (54.5)	13 (61.9)	24 (63.2)	0.742
Status epilepticus, n(%)	2 (6.1)	5 (23.8)	5 (13.2)	0.168
No documented reason for transportation, n(%)	16 (48.5)	4 (19.0)	8 (21.1)	0.019
**Initiated therapy**				
Patients with administered medication (without liquids), n(%)	5 (15.2)	9 (42.9)	20 (52.6)	0.004
if administered (median (IQR))	1 [1, 2]	1 [1, 2]	1 [1, 2]	0.874
Glucose, n(%)	0 (0.0)	1 (4.8)	1 (2.6)	0.489
Induction of anesthesia & intubation, n(%)	1 (3.0)	2 (9.5)	2 (5.3)	0.231

Data are median and interquartile range or n. Reference for sex is female. IQR, Interquartile Range; GCS, Glasgow-Coma-Scale; EMS, Emergency Medical Service. “Pre-existing cerebral damage” includes brain tumor and metastases, intracranial hemorrhage and malformations as well as infantile brain damage and stroke; CI, Confidence Interval; Logistic regression was calculated for univariate significant variables for patients without substance abuse or without status epilepticus, odds-ratios and p values are presented.

## Discussion

In this analysis of prehospital seizures, the study was able to show that more than 90% of the patients were transported to hospital. Several findings support the hypothesis that transportation and hospital admission can be considered unnecessary in many of these cases: in almost 40% no indication for transport was documented by the prehospital emergency physician and a large part of these patients were treated solely in the outpatient department. Additionally, the neurological condition was only mildly impaired at EMS arrival and GCS had increased to a median of 15 in patients with a history of seizures/epilepsy by the time they arrived to hospital. Older patients and patients with higher amounts of administered medication at the scene were hospitalized for more than 24 h, significantly more often. GCS was adequate in most cases at the time of EMS arrival indicating that seizures might have already ceased, questioning the necessity of hospital admission for these patients. The prevalence of seizures, of 4.9% out of all investigated emergency medical services records, was comparable to other studies stating incidences of 3–5%^1–3^. As there are no official guidelines for treating seizures in prehospital emergency medicine in Germany, the UK guidelines were used as an example to assess the necessity of hospital admission^7^. Following these guidelines, patients, who have a history of epilepsy and make a full recovery, are not at risk and if they can be supervised adequately could be treated at home. Also the professional association of head emergency physicians in Germany states, that patients with a history of epilepsy with full recovery under sufficient supervision could be treated at home^15^. In contrast, history of seizures had no influence on transport, hospitalization or outpatient care in our analysis. A potential explanation for this may be the absence of guidelines and, consequently, the lack of confidence among EMS professionals in the treatment of out-of-hospital seizures in Germany.

Above that, our transportation rates were equally high compared to other studies from the UK (70%) and Germany (87%), although in 53% no medication was given^1,2^. There are several possible reasons: Sherratt et al. revealed the need for more training in seizure management as paramedics stated seizure management being neglected in training and lacking feedback, whether transporting patients to an emergency department was indicated^11^. Above that, ambulance funding in the UK underlies time-based targets. Non-conveyance extends time ‘on-scene’ and consequently, precludes acquisition of subsequent calls with eventual penalization of the service^11^. Likewise, Burrell et al. showed a lack of confidence in training and guidance for treating seizures. Only 1/3 of the interviewed ambulance clinicians felt confident in managing seizures. Further management was based more on experience than on training and guidelines emphasizing the development of a tailored training program for emergency staff^12^ This is similar to this study, where documented reasons were heterogenous or missing. The high rate of outpatient care or hospitalization for less than 24 h as well as the only mildly impaired neurological condition at hospital handover supported this finding. A potential explanation for this may be the absence of guidelines and, consequently, the lack of confidence among EMS professionals in the treatment of out-of-hospital seizures in Germany. To date, there are no comparable studies evaluating training and confidence of German emergency physicians or paramedics. Responsibility and the decision about hospitalization often lies upon emergency physicians in the case of altered consciousness rather than paramedics as indicated by the Bavarian emergency service law. However, a median GCS of 14 at arrival indicated only a mildly impaired neurologic state, not justifying the presence of an emergency physician or admittance to an emergency department in the first place.

These findings are in line with Tonn et al. with a median GCS of 13 and transportation rate of 87%. The authors reported, that less then 50% of the emergency calls for "cerebral seizure event" had clinical symptoms, which required medical treatment at the scene. They concluded, that a routine emergency physician alert is only justified with additional parameters indicating severity (e.g. after first GCS classification by paramedics)^2^. Such unnecessary emergency physician alerts and utilization of hospital resources may have a huge economic impact. Dickson et al. suspected the cost of management of suspected seizures at around 67 million U$/year^1^. Elderly patients and patients with complex initial care at the scene were significantly more likely to be hospitalized for a longer period of time. It is likely that in these cases the seizure represents the clinical manifestation of another severe underlying condition (for example, cerebral hemorrhage, stroke, or tumor disease), since a new diagnosis of epilepsy in older age is uncommon. These factors (age, complex initial care and medication) could allow for additional risk stratification at the site and might reduce hospitalization rates and costs.

## Strength and limitations

The strength of this analysis is the large number of evaluated emergency records over a long time period and the homogenous staffing of the EMS by one hospital. However, this study has some limitations: most importantly, there are no detailed standard operating procedures for our site on how to proceed in the event of a prehospital seizure. The approach is left to the emergency physician in charge. However, it is precisely this factor that led to the idea for this analysis and will result in the creation of guidelines as a consequence of our findings. Due to the retrospective analysis information is limited to the documented routine records. This consequently implies that the patients' information on the history of epilepsy as well as medication or previous diagnostics could not be cross-checked using hospital documents. Whether patients could have been adequately monitored at home has been documented in a few cases only. That transport was provided in order to not leave patients unattended at the scene cannot be excluded. As we cannot distinguish between calls in public areas or at home, the decision to transport may be influenced by the requirement to leave the patient in a safe environment. Second, follow-up was only feasible, when patients were transported to one specific university hospital. This led to a high number of patients without further information about their trajectory and diminishes the significance of our results, considering the initial high number of deployment protocols sighted. This is especially true for patients who were left at the scene and not transported to the hospital. With regard to the secondary outcome of length of hospital stay, this therefore remains merely a monocentric study. Characteristics of the patients transported to our hospital, however, did not differ from patients transported to other hospitals. That sub-cohort can therefore be considered representative. In addition to variation in EMS physician practice in the prehospital setting, there is physician practice variation at our hospital with regard to discharge or admission as this is always also a subjectively influenced decision. In addition, the decision to select a hospital length of stay greater than 24 h as a secondary endpoint may also be questioned. However, in our hospital there is no shorter interval in which a patient is discharged home should there be no recurrent epileptic activity. In the case of a clinically well patient, discharge home occurs after completion of the diagnostic workup and not at a pre-defined time period.

This likely introduces confounding for the results of the study. Again, however, it is this depiction of a real-world practice that is part of the result of this study and underscores the necessity of creating guidelines in our EMS system. This will allow for future analyses in which patient outcomes can be prospectively and robustly evaluated.

## Conclusion

There are no official guidelines for emergency physicians in Germany, to advise whether transportation of patients with seizures in prehospital emergency service is indicated. British guidelines were therefore used as an example for comparison purposes. The data from this study is in keeping with previous studies and showed high rates of transportation to hospital without a documented medical reason. History of seizures had no influence on hospitalization. Elderly patients with complex initial care at the scene were significantly more likely to be hospitalized for a longer period of time. This should be considered as important for risk stratification. This data suggests that most hospital transportations in our EMS system were not indicated since the majority were treated as outpatients only. Evidence-based guidelines might facilitate decisions for emergency physicians at the scene, leading to a more uniform approach. This may prevent distress for patients and their caregivers, and may reduce healthcare costs.

## Methods

### Study design

We performed a retrospective analysis of all EMS records from 01/2014 to 12/2016 at the EMS location of Munich—Riem, Germany in order to examine the reasons for hospitalization of patients with seizures and their treatment. This analysis is covered by the ethical approval 508/16 of the ethical committee of the Technical University of Munich (Ethikkommission der Technischen Universität München, Munich, Germany) and was performed under the declaration of Helsinki. Due to the retrospective design of the study and the anonymization of the data, the Ethics Committee waived the need to obtain written informed consent.

### Study setting

The physician staffed EMS location in Munich—Riem is one of ten emergency departments in the greater Munich area. The EMS was provided by 60 emergency physicians (EP), all employed by the university hospital “Klinikum Rechts der Isar” and is manned 24/7. All physicians have the additional qualification “prehospital emergency medicine”. 46 of the physicians were specialized in anesthesiology, 9 in surgery and 1 in internal medicine. They are accompanied by emergency paramedics staffed by the professional fire department of the city of Munich.

### Participants

We screened all available records completed by emergency physicians for relevant seizures. These are available in handwritten form and were not digitalized at the time of the analysis apart from accounting-relevant factors. Exclusion criteria were a diagnosis other than seizures, interhospital transportation, false alerts and patients younger than 16 years. Of the selected records those without information about transportation were also excluded (n = 19). These records are standardized and developed by the German association for intensive and emergency care (DIVI) and valid for all counties of Germany. In addition to medical and patient-related data these records capture the type of operation, different operating times and information about means and destination of transportation. In addition to the predefined fields, free text is possible and was also analyzed in regards to pre-existing conditions, supplementary information on the seizure, medication given by someone other than the emergency physician and abuse of alcohol or other substances. Furthermore, the suspected diagnosis had to be filled in and served as our main selection criteria.

### Outcomes

The primary endpoint of our study was the number of patients with prehospital seizures transported to hospital, their characteristics and the reason for transportation. Since patients with their first seizure should be assessed at a hospital, they were grouped by known epilepsy and their baseline condition. Patients who were transported to Klinikum rechts der Isar, a university hospital of the Technical University Munich, could be followed up further. No further information is available on patients left at the scene where no hospital admission was made. With regard to the secondary outcome of length of hospital stay, this remains only a monocentric study. Other hospitals could not be assessed to data protection regulation. As a secondary endpoint, we evaluated patients who remained in the hospital beyond a period of initial monitoring (> 1 days) and their length of stay. These were compared with the cohort of patients, who were seen as outpatients or remained in the hospital for less than 1d. Finally, a comparison was made between the transport indications given by the emergency physician and the patients' length of hospital stay.

### Exposures and predictors

All medical parameters, that were available upon arrival of the medical emergency team and potentially influenced the decision whether to transport the patient, were used as predictors. These were gender, GCS at EMS arrival, first occurrence of a seizure, abuse of alcohol and, if the patient was still convulsing or had status epilepticus. Additionally, comorbidities and the circumstances surrounding the seizure such as traumatic brain injury, clinical signs of seizures (e.g. urinary incontinence, generalized seizure, signs of infection) or blood sugar levels requiring the administration of glucose were included into the analysis. The category ‘pre-existing cerebral damage’ was defined as patients with an existing brain tumor or metastases, intracranial hemorrhage and stroke, malformations or prior infantile brain damage. The condition "Traumatic brain injury" represents the reason for calling the emergency medical services. Out-of-hospital treatment was defined as the need for general anesthesia or securing the airway. The number of medications required was recorded. A high amount of administered medication or the need for general anesthesia was defined as complex initial care. In regards to the category ‘benzodiazepine administered’ we assumed an emergency physician to administer these only, if the physician considered the patient at risk for status epilepticus. Additional data about the necessity of hospitalization and the subsequent clinical course was only available for patients who were transported to the university hospital “Klinikum rechts der Isar” because of data protection regulations.

In a second analysis, we examined the trajectory of patients who were admitted to our hospital regarding their hospital length of stay and mortality.

### Statistical analysis

Statistical analyses were performed using R Version 4.0.5. Continuous data are described by median (interquartile range from quartile 25% to quartile 75%), and categorical data by absolute and relative frequencies. Data were analyzed using a chi-square or Mann–Whitney U test for categorical and continuous variables, respectively. A multivariate logistic regression model with all univariate significant variables obtained through medical history and initial survey of the patient was calculated. For comparison of hospital length of stay of transported patients log rank tests were used and presented using Kaplan Meier Curves. A two-sided p value of less than 0.05 was considered statistically significant, correction for multiple testing was performed using Bonferroni method.

## Supplementary Information

Supplementary Tables.

## Data Availability

Data can be obtained from the corresponding author on reasonable scientific request and as long as German data protection law can be complied with. Requests to access the datasets should be directed to Stefan Schaller, s.schaller@tum.de.

## Abbreviations

EMS: Emergency Medical Service
GCS: Glasgow Coma Scale
UK: United Kingdom
EP: Emergency Physician
DIVI: German Association for Intensive and Emergency Care
TBI: Traumatic Brain Injury
IQR: Interquartile Range
LOS: Length of Stay
CI: Confidence Interval
