# Supplementary material for: Excessive hospitalization of patients with seizures in the Germany prehospital emergency system: a retrospective cohort study
Source: Sci Rep. 2022 Jun 27;12:10866. doi: 10.1038/s41598-022-15115-8 (PMC9237077; doi:10.1038/s41598-022-15115-8)
Supplement: Supplementary file 1 — Supplementary Tables. [file 41598_2022_15115_MOESM1_ESM.pdf]

Supplement

Excessive hospitalization of patients with seizures in the Germany prehospital emergency system – a retrospective cohort study

Supplement Table 1 Reasons for hospital transportation for patients with known epilepsy; including overlaps 2

Supplement Table 2 Length of hospital stay for patients with and without documented reason for hospital transportation to Klinikum rechts der Isar, Munich. 2

Supplementary Table 1 Reasons for hospital transportation for patients with known epilepsy; including overlaps

	All patients, n =206
Status epilepticus	27 (13.1%)
Subsequent seizure	18 (8.7%)
Still seizing at EMS arrival	47 (22.8%)
Possible TBI	11 (5.3%)
Preexisting neurological condition	38 (18.4%)
Seizure relating to C2-/ substance abuse	23 (11.2%)
Possible infection / fever	3 (1.5%)
GCS at handover (median [IQR])	
>14	59 (28.6%)
≤14	51 (24.8%)
n/a	96 (46.6%)
Benzodiazepines administered by EMS	67 (32.5%)
No reason documented	76 (36.9%)

Data are n(%). TBI, Traumatic Brain Injury; GCS, Glasgow-Coma-Scale; EMS, Emergency Medical Service. "Pre-existing neurological condition" includes brain tumor and metastases, intracranial hemorrhage and malformations as well as infantile brain damage and stroke. Overlaps are included, i.e. a patient can have multiple reasons for transport. In 37% of the cases no reason was documented by the prehospital emergency physician.

Supplementary Table 2 Length of hospital stay for patients with and without documented reasons for hospital transportation.

LOS Hospital (days)	Reason documented, n= 64	No reason documented, n= 29
0	17 (26.6%)	16 (55.2%)
1	17 (26.6%)	5 (17.2%)
2	3 (4.7%)	1 (3.4%)
3	8 (12.5%)	3 (10.3%)
4	5 (7.8%)	2 (6.9%)
5	2 (3.1%)	0 (0.0%)
6	2 (3.1%)	1 (3.4%)
≥7	10 (15.6%)	1 (3.4%)

Data are n(%). LOS, Length of stay. Median of LOS for all patients is 1.0 [IQR 1 to 2], median for patients without documented transportation reason is 0 [IQR 0 to 1] days.
